# The Use of California Sagebrush (*Artemisia californica*) Liniment to Control Pain

**DOI:** 10.3390/ph5101045

**Published:** 2012-09-25

**Authors:** James D. Adams

**Affiliations:** Department of Pharmacology and Pharmaceutical Sciences, School of Pharmacy, University of Southern California, 1985 Zonal Avenue, Los Angeles, CA 90089, USA; Email: jadams@usc.edu; Tel: +1-323-442-1362

**Keywords:** *Artemisia californica*, California sagebrush, pain therapy

## Abstract

The incidence of arthritis is increasing every year, as does the need for pain medication. The current work reviews an American Indian liniment that is traditionally used for pain therapy. The chemistry, therapeutic use and safety of the liniment are reviewed. The liniment contains monoterpenoids, sesquiterpenes, flavonoids, alkaloids and other compounds.

## 1. Introduction

The Centers for Disease Control provide statistics that show the incidence of osteoarthritis is increasing every year. This parallels increases in obesity and metabolic syndrome every year. Obesity increases the body’s burden of toxic lipids such as ceramide and the endocannabinoids [1]. These toxic lipids increase the release of inflammatory adipokines from visceral fat that lead to joint inflammation, osteoarthritis and pain [1]. Lifestyle changes, such as losing weight and daily exercise could decrease the incidence of arthritis [1]. Modern medicine could work more effectively with patients to promote lifestyle changes. However, there is always a need for pain medications. Many medications are available to modify the disease process in arthritis. However, all of them suffer from toxicity problems such as ulcers and kidney toxicity.

## 2. *A. californica* Liniment

A traditional Liniment for use in pain patients is described here. The liniment is easily used by topical application and can be used in any clinical setting. Relief of pain is rapid, usually within 20 minutes and can last 2–3 hours even in severe pain patients. Reapplication of the liniment results in successful pain therapy in most patients. The alcoholic liniment is made from *Artemisia californica* (*A. californica)* by California Indians [2]. Traditionally, the liniment was made from *A. californica* and bear grease or whale oil. The stems and leaves of the plant are soaked in alcohol, such as isopropanol or ethanol, for several weeks in the dark. A leaf of *Salvia apiana* and several seeds of *Pursea americana* are added as well. The liniment is used by application of small amounts to the skin in the areas where the pain is greatest. *A. californica* liniment has been used anecdotally in many patients with successful pain relief in every patient [3]. These pain patients suffered from various ailments such as arthritis, muscle and ligament strains, bruises, broken bones, low back pain and cancer.

## 3. Chemistry of *A. californica*

**Figure 1:**
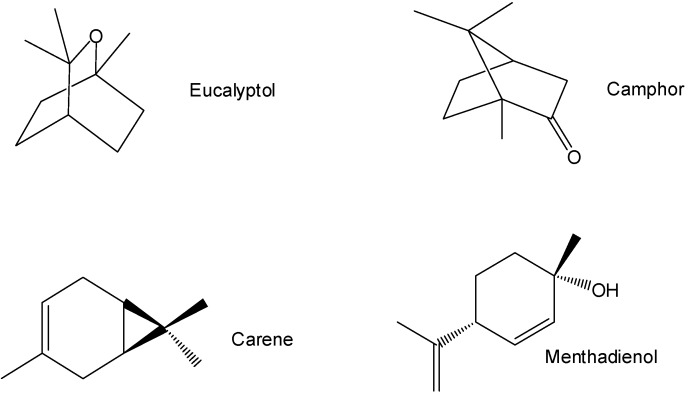
The major monoterpenoids in *A. californica*.

A recent study [3] of the chemistry of *A. californica* found 15 monoterpenoids: camphene, mentha-diene, β-pinene, eucalyptol, isopropenylmethylcyclohexanol, trimethylheptadienol, isopropylmethyl-bicyclohexanol, thujanone, thujone, chrysanthenone, camphor, borneol, carene, menthenol and menthadienol [3]. These products were identified by the molecular ions and fragmentation ions obtained by gas chromatography/mass spectrometry. The major monoterpenoids (Figure 1) were eucalyptol (24%), camphor (18%), carene (14%), and menthadienol (9%).

High pressure liquid chromatography/mass spectrometry (HPLC/MS) found a number of sesquiterpenes, alkaloids, flavonoids and other compounds. These products were identified by their molecular ions, fragmentation ions and UV spectral characteristics. The major compounds found by HPLC/MS were: jaceosidin, 6-methoxytricin, chrysosplenetin, and quercetin hexose, all flavonoids [3]. Other flavonoids found were: luteolin gentiobioside, tamarixetin (glycoside), isoorientin and methoxyflavone hexose [3]. Alkaloids found were usaramine, rupestine, and chromonar [3]. Sesquiterpenes found were leucodin, pestalodiopsolide A, echinolactone B, tanapartholide A and secogorgonolide [3]. An anthocyanin, apigeninidin glucoside, was also found [3]. A coumarin found was marmin [3] and a chalconoid (monoterpenoid) found was xanthohumol disaccharide [3].

## 4. Pharmacology of the Monoterpenoids from *A. californica*

The monoterpenoids found in *A. californica* are powerful pain relievers that inhibit transient receptor potential cation channels (TRP). These channels are located in sensory neurons of the skin, brain stem, brain, lungs and other areas [4]. TRP channels also react to cold or hot temperatures. Opening of a TRP channel usually allows sodium and calcium to enter the neuron. The pain cycle involves an initial stimulus of the pain, usually in skin sensory neuron TRP channels, transmission of the stimulus to the spinal cord, modulation of the stimulus in the spinal cord and brain, and perception of the pain in the brain [5]. Perception of the pain may cause a reflex increase in the activity of the sensory neurons of the skin and hence more pain. Breaking the pain cycle in the skin can be easily accomplished with skin penetrating monoterpenoids. These compounds can be applied as a liniment where they are needed in small amounts. This avoids systemic administration of large amounts of pain relievers. The monoterpenoids are rapidly cleared from the skin and have little toxicity since they are present only in small amounts.

Monoterpenoids with known pain relieving activity in *A. californica* are camphor [6,7,8], eucalyptol [7,8,9], camphene [7,8], β-pinene [7,8,9], borneol [7,8,10] and thujone [11]. Most of these monoterpenoids penetrate the skin including β-pinene [12], so that they can act topically.

Monoterpenoids are antinociceptive since they bind to TRPV1 (TRP vanilloid1), TRPV3 and TRPM8 (TRP melastatin8) receptors. TRPV1 and 3 are important in nociception and thermosensing [13]. These receptors are found in sensory neurons [14] of the skin, in keratinocytes, in other organs, and in pain pathways such as the dorsal root ganglia, trigeminal neurons, and the spinal cord [13]. TRPM8 is found in most cold-sensitive afferents of the skin and other organs [15]. Monoterpenoids are usually agonists for TRP channels, and can cause transient pain. They quickly deactivate TRP channels, causing long term pain relief.

Most of the pain relieving monoterpenoids found in *A. californica* are agonists for TRPV3 (heat-sensitive) including camphor [6,13,16], borneol, thujone and eucalyptol [16]. Camphor is also an antagonist for TRPA1 (TRP ankyrin-repeat1, cold-sensitive) and an agonist for TRPV1 (heat-sensitive) [6]. Eucalyptol is also an agonist for TRPM8 (cold-sensitive, [15]) and has antinociceptive activity comparable to morphine. Morphine and eucalyptol act synergistically and produce much greater than expected pain relief when used together [9].

Anti-inflammatory properties are prominent for some monoterpenoids such as camphene, borneol and β-pinene [17,18,19]. This anti-inflammatory activity is due to inhibition of nitric oxide (NO) and prostaglandin E2 (PGE2) production. The mechanism involves increased expression of IKK (inhibitor of NF-κB kinase), iNOS (inducible nitric oxide synthase), and NF-κB (nuclear factor κB), and decreased expression of IκBα (inhibitor of NF-κBα) [18,19]. It is not known if monoterpenoids applied to the skin have anti-inflammatory activity in arthritic joints. This needs to be examined.

Oral toxicity of monoterpenoids involves seizures from camphor [20,21], thujone [20,22] and camphene [20]. Anti-convulsant activities are reported for oral β-pinene, eucalyptol [23] and borneol [10]. Monoterpenoids, in essential oils, applied to the skin can cause skin irritation. However, skin penetration of monoterpenoids in quantities sufficient to cause convulsions or other toxicity has not been reported, except in infants.

## 5. Pharmacology of the Flavonoids from *A. californica*

**Figure 2:**
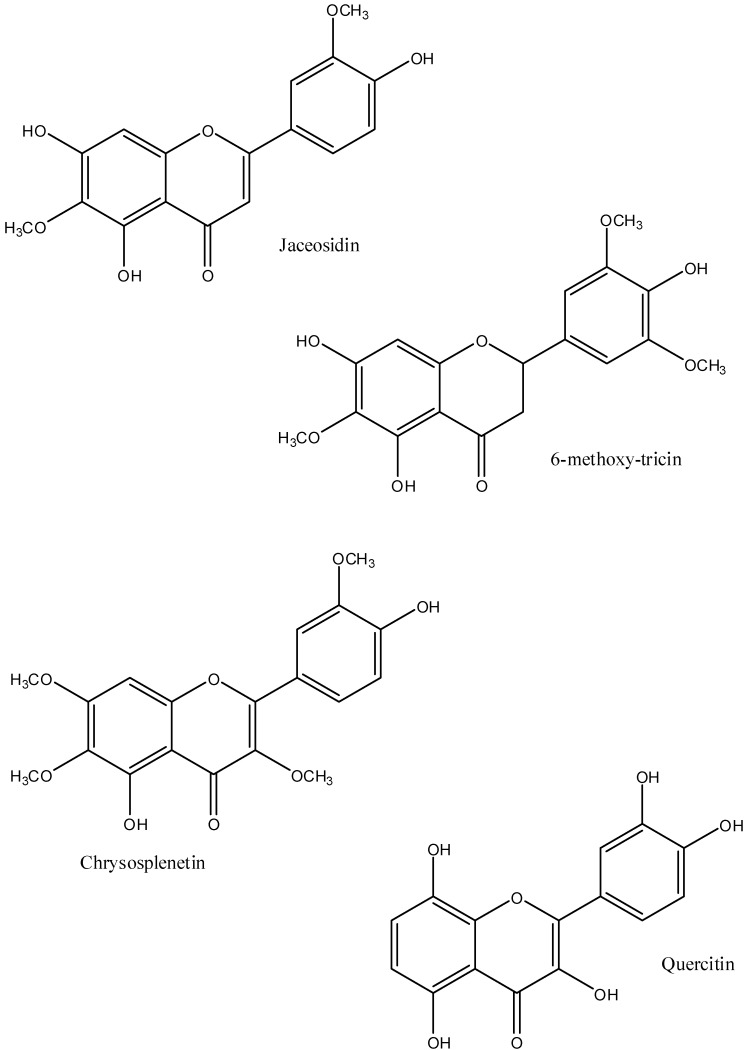
The major flavonoids in *A. californica*. Quercitin hexose has a hexose linked to the 4' hydroxyl group of quercitin.

Several flavonoids have anti-inflammatory and analgesic activities. 6-Methoxytricin (Figure 2) is anti-inflammatory and inhibits T cell proliferation and activation [24]. Quercetin and quercetin glycoside are anti-inflammatory and decrease tumor necrosis factor alpha and NO production [25]. They are also analgesic due to serotonin 5-HT1A receptor activation [7,8]. Jaceosidin is anti-inflammatory, penetrates the skin and relieves inflammation by inhibition of the induction of NFkB [26]. These flavonoids clearly add to the analgesic and anti-inflammatory effects of *A. californica* liniment and may act synergistically with the monoterpenoids.

## 6. Pharmacology of the Alkaloids from *A. californica*

**Figure 3:**
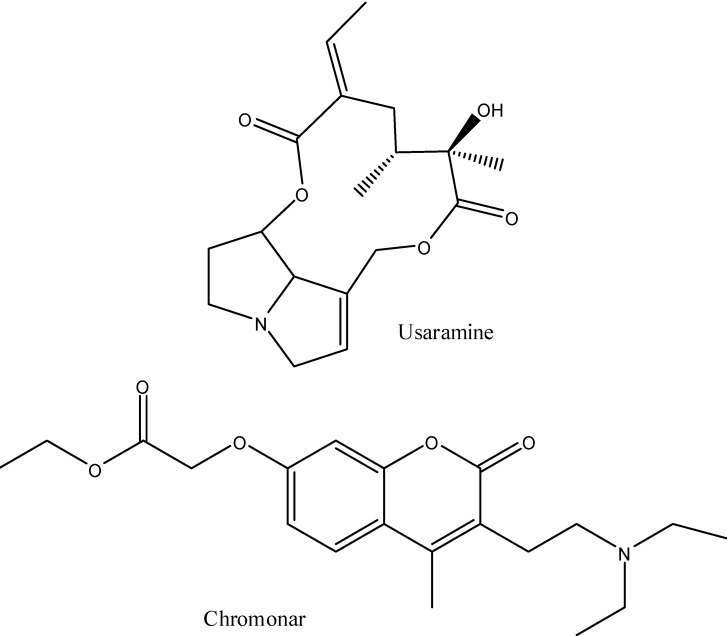
The major alkaloids in *A. californica*.

Usaramine is a macrolide pyrrolizidine alkaloid (Figure 3). Pyrrolizidine alkaloids that can be converted by the liver into pyrroles are toxic and carcinogenic [27]. However, usaramine has not been reported to be toxic, carcinogenic or to be converted into a pyrrole. It is present in several medicinal plants and may be anti-inflammatory. Chromonar has a chloro analog, cloricromene, that does not appear to be useful for relief of arteriopathy in intermittent claudication. Cloricromene may be able to decrease tumor necrosis factor alpha levels and could be useful in arthritis. The alkaloids in *A. californica* have not been investigated to see if they have pain relieving activity. Other alkaloids, such as scopolamine, are powerful pain relievers and can cross the skin.

## 7. Pharmacology of the Sesquiterpenes from *A. californica*

**Figure 4:**
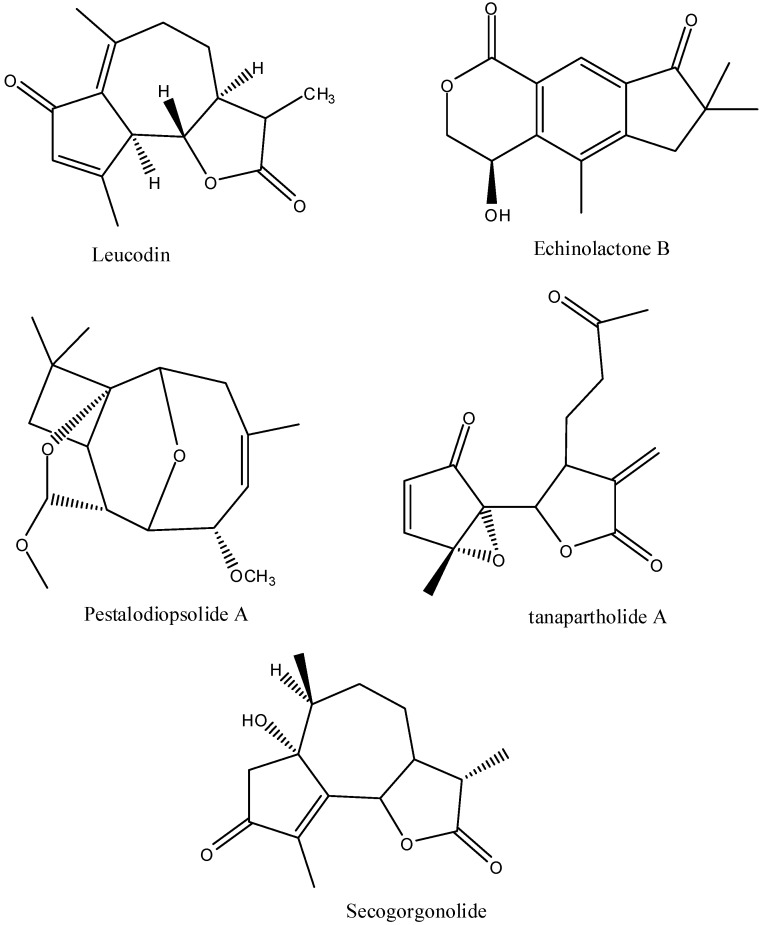
The major sesquiterpenes in *A. californica*.

Sesquiterpenes can have anti-inflammatory activity [17], cross the skin and inhibit an NF-κB pathway (Figure 4). They can also inhibit NO and PGE2 production, inhibit the expression of IL-6, tumor necrosis factor alpha and cyclooxygenase-2 [28]. Leucodin is available as a drug in Brazil for the treatment of skin disorders. Its dehydro form, dehydroleucodine, inhibits peroxisome proliferator activated receptor gamma [29] and is anti-inflammatory [30]. Iso-seco-tanapartholides are anti-inflammatory through an NF-κB pathway [31]. None of the sesquiterpenes in *A. californica* have been tested for pain relieving activity or the ability to cross the skin.

## 8. Pharmacology of the Other Compounds from *A. californica*

Marmin protects gastric mucosa by maintaining the mucosal barrier and by inhibiting the actions of acetylcholine and histamine [32]. Apigeninidin has reported pharmacological activity but is a permanently charged compound that may not cross the skin. Xanthohumol inhibits cyclooxygenase 1 and 2, NF-κB and is therefore an anti-inflammatory agent [33,34]. It is not clear if the disaccharide from xanthohumol disaccharide is cleaved in the skin to allow xanthohumol to cross the skin.

## 9. Conclusions

Native Americans have traditionally used sagebrush liniments in pain therapy. *A. californica* liniment is a complex medicine that contains monoterpenoids, flavonoids, sesquiterpenes and other compounds with anti-inflammatory and pain relieving activity. The main pain relieving targets are the various TRP channels in sensory neurons of the skin. Since there are 15 monoterpenoids that inhibit several different TRP channels, it is very possible that these compounds interact to produce more than additive effects on pain relief. Many of the anti-inflammatory compounds in *A. californica* inhibit NF-κB pathways. Since there are several NF-κB pathway inhibitors in the plant, it is possible they interact to produce more than additive anti-inflammatory effects. It is very likely that *A. californica* liniment is superior to menthol liniment or other liniments made from single purified compounds. Clinical use of the liniment results in satisfactory pain relief in most patients. However, reapplication is required in some very severe pain patients. The use of very small amounts of the topically applied liniment is superior to orally administered agents that must be used at high doses and may adversely affect many organs in the body.
